# Recovery rate and prognostic factors of peripheral facial palsy treated with integrative medicine treatment: a retrospective study

**DOI:** 10.3389/fneur.2025.1525794

**Published:** 2025-03-18

**Authors:** Bonhyuk Goo, Jung-Hyun Kim, Jinkyung Park, Yong-Hyeon Baek, Sang-Soo Nam

**Affiliations:** ^1^Department of Acupuncture and Moxibustion, Kyung Hee University Hospital at Gangdong, Seoul, Republic of Korea; ^2^Department of Clinical Korean Medicine, Graduate School, Kyung Hee University, Seoul, Republic of Korea; ^3^Department of Acupuncture and Moxibustion, Kyung Hee University College of Korean Medicine, Kyung Hee University Hospital at Gangdong, Seoul, Republic of Korea

**Keywords:** Bell’s palsy, Ramsay-Hunt syndrome, House-Brackmann facial grading system, integrative medicine, prognostic factor, recovery

## Abstract

**Introduction:**

This study aimed to identify prognostic factors and develop a classification model for predicting recovery in patients with peripheral facial palsy.

**Methods:**

Data from patients who received integrative medicine treatment with Bell’s palsy and Ramsay-Hunt syndrome were collected. The change of House-Brackmann Grade (HB Grade) for 2 years from the onset and the factors presumed to be related to the prognosis were analyzed by reviewing electrical medical records retrospectively. The estimated recovery rates to HB Grade 2 and 1 were calculated by the Kaplan–Meier method. The factors affecting the prognosis were selected by using univariate Cox regression analysis. Subsequently, multivariate Cox regression analysis was performed on the selected factors. The factors derived from the Cox regression model were applied to the survival tree analysis model to establish the criteria for the classification of patients according to prognosis.

**Results:**

768 participants were included after screening. Based on the Kaplan–Meier method, the estimated recovery rates for HB Grade 2 and 1 for the 2 years were 98.2 and 83.3%, respectively. The univariate Cox regression analysis indicated that ten factors, including sex, diabetes, hemoglobin A1c, diagnosis, periauricular pain, hearing impairment, taste disorder, initial HB Grade, and average axonal loss (AAL) and maximum axonal loss (MAL) of nerve conduction study (NCS), affected prognosis. Finally, multivariate Cox regression showed that the AAL and MAL were related to prognosis. Five classification models predicting the 2-year estimated recovery rate established from the survival tree analysis were as follows: 100% (AAL < 70% and MAL < 80%), 87.1% (AAL < 70% and MAL ≥ 80%), 86.8% (70% ≤ AAL < 80%), 55.0% (80% ≤ AAL < 90%), and 24.2% (AAL ≥ 90).

**Conclusion:**

The present results demonstrated that AAL and MAL of the NCS were significant factors in predicting the prognosis of peripheral facial palsy.

## 1 Introduction

Peripheral facial palsy (PFP) refers to a lower motor neuron lesion of seventh cranial nerve, also known as the facial nerve, and results from facial nerve dysfunction due to trauma or inflammation of the facial nerve or its branches along its course, primarily in the bony canal (1, 2). Partial or complete inability to automatically move the affected side of the facial muscles is the typical symptom of PFP. It can be accompanied by other symptoms, including periauricular pain, hearing impairment, and taste disorder (3–5).

Bell’ palsy (BP), an idiopathic facial nerve palsy, is the most common diseases that cause PFP (6). BP occurs in 11.5 to 53.3 cases per 100,000 population, and 60–75% of PFP patients are BP (4, 7). Ramsay-Hunt syndrome (RHS), which is caused by reactivation of the varicella-zoster virus at the geniculate ganglion, occurs in 5 cases per 100,000 people. RHS has symptoms that do not occur in BP, such as rash, hearing loss, and dizziness, but it has many similarities with BP regarding pathophysiology and clinical management (8–11). In prognosis, BP is usually resolved within weeks or months; However, approximately 25% of patients are reported to suffer from moderate-to-severe facial asymmetry (12) and various types of sequelae, including synkinesis, hemifacial spasm and contracture (13, 14). Moreover, RHS shows a worse prognosis than BP (15, 16).

Incomplete recovery and permanent sequelae of PFP can significantly affect not only the subjective discomforts but also the appearance of the face, leading to lower quality of life and social or psychological problems (17). Furthermore, depending on the severity of the disease, the treatment period can be prolonged for more than 1 year, and the patients can experience severe anxiety and depression over time during the treatment course (18). Therefore, accurate prediction of the prognosis of PFP plays a crucial role in establishing a treatment plan early in the acute phase and in providing psychological stability to patients on clinical sites (19).

Several studies have identified some predictive factors for prognosis in PFP, including patient characteristics, underlying diseases, severity of disease, accompanying symptoms, and electrophysiological examinations. Most of the studies have found that early assessments of severity using the House-Brackmann Grade (HB Grade), Yanagihara scale, and nerve conduction study (NCS) are associated with the prognosis of facial paralysis (20–23). In addition, there have been reports that age (24, 25), hypertension (HTN) (24), and diabetes mellitus (DM) (26) are related to the prognosis of PFP.

Integrative medicine treatment (IMT) is a healthcare approach that combines modern conventional medicine with complementary and alternative medicines. There has been increasing interest in multidisciplinary approaches aimed at improving the recovery of paralysis and enhancing patients’ quality of life of PFP (27). In Korea, IMT is mainly conducted as a combination of modern conventional medicine and traditional Korean medicine (TKM). According to a report based on national health insurance data, among the 700,415 Bell’s palsy patients from 2002 to 2018, 453,447 patients (64.9%) received only TKM treatment. 133,906 patients (19.1%) received IMT, and this proportion is increasing (28).

As conservative conventional treatments, using corticosteroids, antiviral agents, and physical therapy is suggested as a standard treatment approach in guidelines and studies (29–33). The clinical practice guidelines for facial palsy in TKM suggest various treatment methods, including manual needle acupuncture, electroacupuncture, thread-embedding acupuncture, pharmacopuncture, moxibustion, herbal medicine, and cupping (34). Based on this evidence, IMT for PFP has been applied in Korea as a standardized program (35, 36). Despite the high preference and utilization of IMT for PFP, large-scale studies on the treatment effects or prognostic factors for patients receiving IMT are lacking.

In this study, we investigated the effectiveness of IMT in patients with PFP and identified the factors affecting the prognosis of PFP. Then, based on the derived factors, we suggested a clinical classification model of PFP for predicting the prognosis and the detailed recovery rate over time.

## 2 Materials and methods

### 2.1 Study design

This study collected and analyzed the clinical data of the patients from the electrical medical record (EMR) in the retrospective method. Using survival analysis methods, the factors affecting the recovery of PFP within 2 years from onset were analyzed, and the estimated recovery rate based on the classification by those factors was calculated. The protocol of this study has been approved by the Institutional Review Board of Kyung Hee University Korean Medicine Hospital at Gangdong (Registration number: KHNMCOH-2013-03-016-004), and the methodology was established in accordance with the Strengthening the Reporting of Observational Studies in Epidemiology (STROBE) guidelines.

### 2.2 Participants

**Figure 1:**
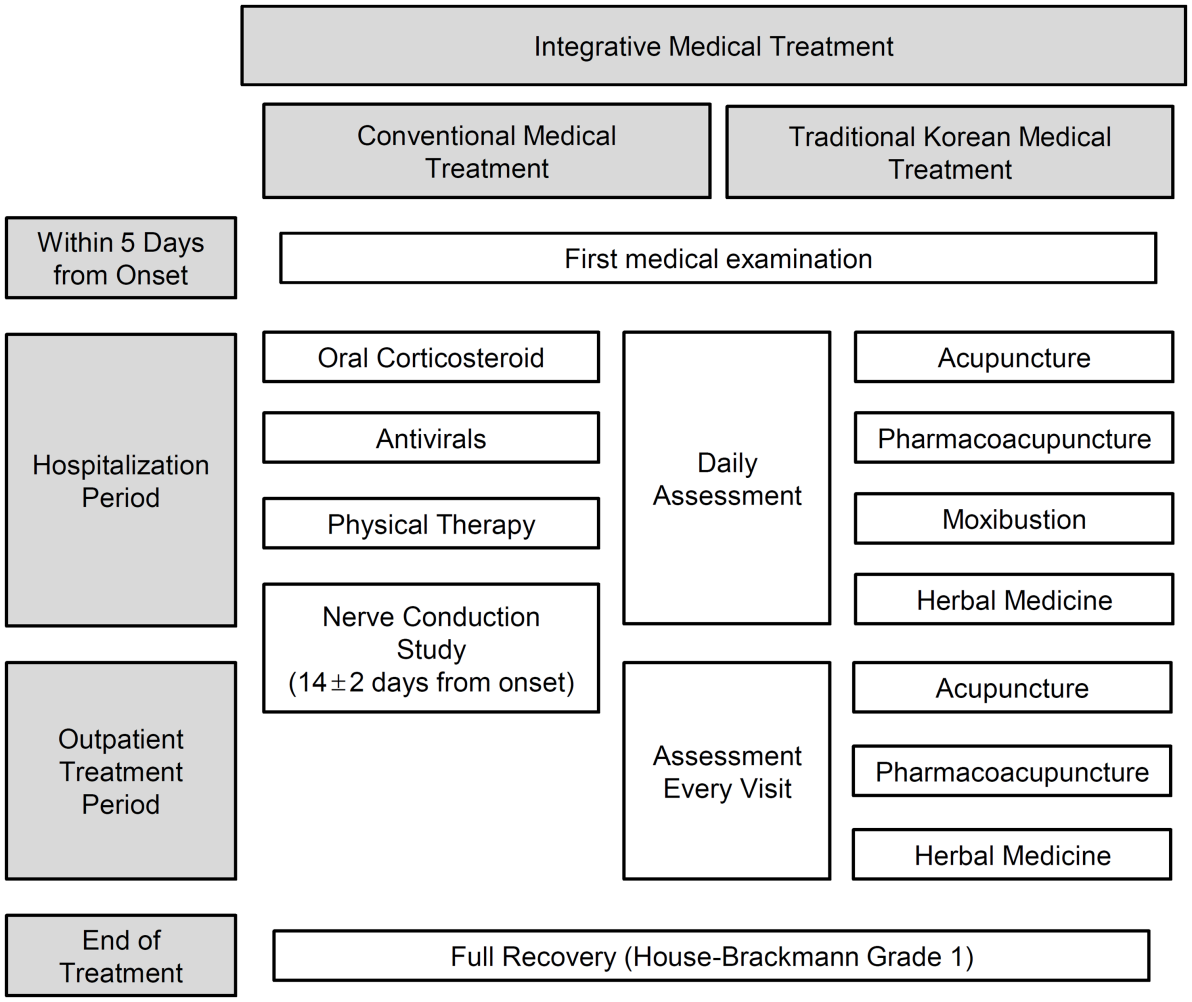
The procedure of the integrative medical treatment.

The present study included patients with BP or RHS who visited the Facial Palsy Center in Kyung Hee University Hospital at Gangdong from June 1, 2006, to December 31, 2014, and received IMT (Figure 1). BP was initially diagnosed based on clinical symptoms and medical history. Then secondary causes such as central, infectious, traumatic, space-occupying, and autoimmune causes were ruled out through diagnostic tests (37). RHS was diagnosed based on symptoms of facial weakness accompanied by vestibular rashes around the ears, hearing loss, or vertigo (38, 39).

The exclusion criteria were as follows: (1) patients who visited the hospital 6 days after onset, (2) patients under 18 years of age (40), (3) recurrent facial palsy, (4) bilateral facial palsy, (5) pregnancy (41), and (6) no NCS record.

### 2.3 Experimental settings

Participants who met the eligibility criteria were selected and their medical records within 2 years from onset were reviewed through the EMR system. Data associated with the patients including demographics, medical history, initial symptoms, treatment period, severity assessment, and examinations were collected using data extraction forms. All procedures were performed and cross-checked by two independent researchers. Private information unrelated to the study was not collected, and personally identifiable information was discarded after analysis.

### 2.4 Variables

#### 2.4.1 Demographics and medical history

Factors about demographics and medical history including age, sex, and history of HTN and DM were collected. Level of glycosylated hemoglobin (HbA1c), which shows patients’ glycemic control over 2–3 months (42), was examined in the first medical examination. Additionally, the period from the onset to the first visit, hospitalization period, and total follow-up period were calculated.

#### 2.4.2 Diagnosis

Information about the palsy side and the accompanying symptoms, including periauricular pain, hearing impairment, and taste disorder, were reviewed from the initial medical records. The presence of each symptom was recorded by asking questions about subjective discomfort.

#### 2.4.3 The House-Brackmann Grade

The HB Grade was assessed daily during the hospitalization periods and every visit during the outpatient treatment periods. The HB Grade evaluates the severity of a patient’s facial palsy into six grades: 6 (total paralysis), 5 (severe dysfunction), 4 (moderate severe dysfunction), 3 (moderate dysfunction), 2 (mild dysfunction), and 1 (normal). Generally, HB Grade 2 usually implies good recovery, and the HB Grade 1 suggests complete recovery (43). In order to reduce the deviation of assessment, periodic training and meetings were conducted.

The most severe grade of each participant in the acute stage was used as a factor that indicates apparent severity. As primary outcome measures, whether the recovery to HB Grade 1 was completed and its period were used as the time and status variables in the survival analysis.

#### 2.4.4 The NCS assessment

We conducted NCS for the facial nerve 14 (±2) days after the onset of PFP. The compound muscle action potential (CMAP) amplitude of both sides was measured in the muscles of the frontalis, orbicularis oculi, nasalis, and orbicularis oris. Then, the axonal loss was calculated at four sites according to the following equation:


$$\text{Axonal}\;\text{loss}\left(\%\right)=\frac{\begin{array}{l} \text{CMAP}\;\text{amplitude}\;\text{of}\;\text{normal}\\ \;\text{side}-\text{CMAP}\;\text{amplitude}\;\text{of}\;\\ \text{affected}\;\text{side} \end{array}}{\text{CMAP}\;\text{amplitude}\;\text{of}\;\text{normal}\;\text{side}}\times 100$$

The average and maximum values of four axonal losses were used for a factor that indicates electrophysiological degeneration of the facial nerve. For the analysis, the average axonal loss (AAL) and maximum axonal loss (MAL) were divided into five groups of 60, 70, 80, and 90% points as cut-off points, respectively.

### 2.5 Sample size

**Figure 2:**
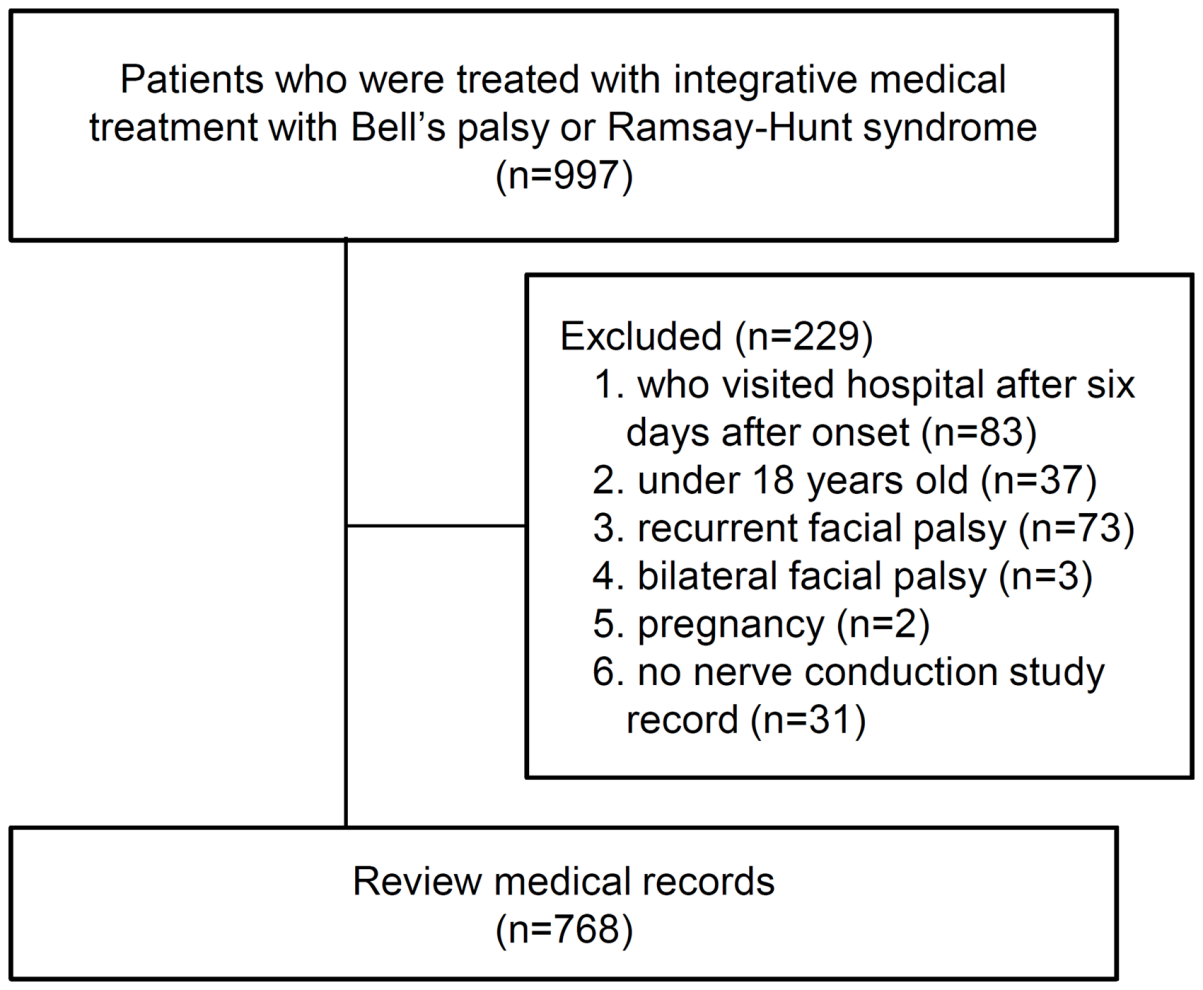
Study flowchart.

A total of 997 patients with BP or RHS who received IMT were retrieved by searching the diagnostic codes and admission records. Subsequently, 229 patients were excluded according to the exclusion criteria. Finally, 768 participants were included in the final analysis, and their medical records were reviewed (Figure 2).

### 2.6 Statistical methods

If there were records of visits without assessment records, the missing records of the HB Grades were corrected by using the last observation carried forward (LOCF) method. The 2-year recovery rate to HB Grade 2 and 1 was calculated for all participants using the Kaplan–Meier curves. In order to calculate the factor associated with the recovery of PFP, multivariate proportional hazard Cox regression analysis was performed for variables selected by univariate analysis (*p* < 0.2). Subsequently, survival tree analysis was performed on the factors derived from the Cox regression analysis, and the Kaplan–Meier curves were calculated for each node of the survival tree. The Kaplan–Meier curve and Cox regression analysis were performed using PASW Statistics 18 (SPSS Inc., Hong Kong, China), while the survival tree analysis was performed using the conditional inference tree (CTree) in the “partykit” package of R (The R Foundation for Statistical Computing, Vienna, Austria) (44). The cut-off value for statistical significance was set at *p* = 0.05.

## 3 Results

### 3.1 Characteristics of the participants

The study included 768 participants (346 men and 422 women) with an average age of 49.69 ± 13.96 years. The proportion of patients with HTN and DM was 21.5% (*n* = 165) and 15.8% (*n* = 121), respectively, and the mean value of HbA1c was 5.77 ± 1.06%. On average, participants visited the hospital for 1.76 ± 1.38 days after onset, received inpatient treatment for 20.19 ± 6.10 days, and continued to receive treatment from the onset up to 96.37 ± 128.85 days in the outpatient clinic.

The diagnosis rate was 91.5% (*n* = 703) in BP and 8.5% (*n* = 65) in RHS. The facial paralysis was on the patient’s left and right side in 51.6% (*n* = 396) and 48.4% (*n* = 372). The most common accompanying symptom was periauricular pain (56.3%, *n* = 432), followed by taste disorder (34.5%, *n* = 265) and hearing impairment (17.1%). The proportion of HB Grades in the acute stage was as follows: Grade 5 (28.5%, *n* = 219), Grade 4 (49.7%, *n* = 382), Grade 3 (19.3%, *n* = 148), and Grade 2 (2.5%, *n* = 19). The mean of AAL and MAL was 60.22 ± 20.69% and 73.49 ± 17.99%, respectively (Table 1).

**Table 1:** Characteristics of the participants.

Characteristics		Unit	Mean ± SD or n (%)
Age		Years	49.69 ± 13.96
Sex	Male		346 (45.1%)
	Female		422 (54.9%)
Hypertension			165 (21.5%)
Diabetes mellitus			121 (15.8%)
Hemoglobin A1c		%	5.77 ± 1.06
Period from onset to visit		Days	1.76 ± 1.38
Hospitalization period		Days	20.19 ± 6.10
Follow-up period		Days	96.37 ± 128.85
Diagnosis	Bell’s palsy		703 (91.5%)
	RHS		65 (8.5%)
Palsy side	Left		396 (51.6%)
	Right		372 (48.4%)
Periauricular pain			432 (56.3%)
Hearing impairment			131 (17.1%)
Taste disorder			265 (34.5%)
House Brackmann Grade On onset	2		19 (2.5%)
	3		148 (19.3%)
	4		382 (49.7%)
	5		219 (28.5%)
AAL	Average	%	60.22 ± 20.69
	AAL < 60%		367 (47.8%)
	60 ≤ AAL < 70%		116 (15.1%)
	70 ≤ AAL < 80%		123 (16.0%)
	80 ≤ AAL < 90%		113 (14.7%)
	AAL > 90%		49 (6.4%)
MAL	Average	%	73.49 ± 17.99
	MAL < 60%		171 (22.3%)
	60 ≤ MAL < 70%		128 (16.7%)
	70 ≤ MAL < 80%		136 (17.7%)
	80 ≤ MAL < 90%		168 (21.9%)
	MAL > 90%		165 (21.5%)

SD, standard deviation; RHS, Ramsay-Hunt syndrome; AAL, average axonal loss; MAL, maximum axonal loss.

### 3.2 Analysis of the 2-years recovery rate

**Figure 3:**
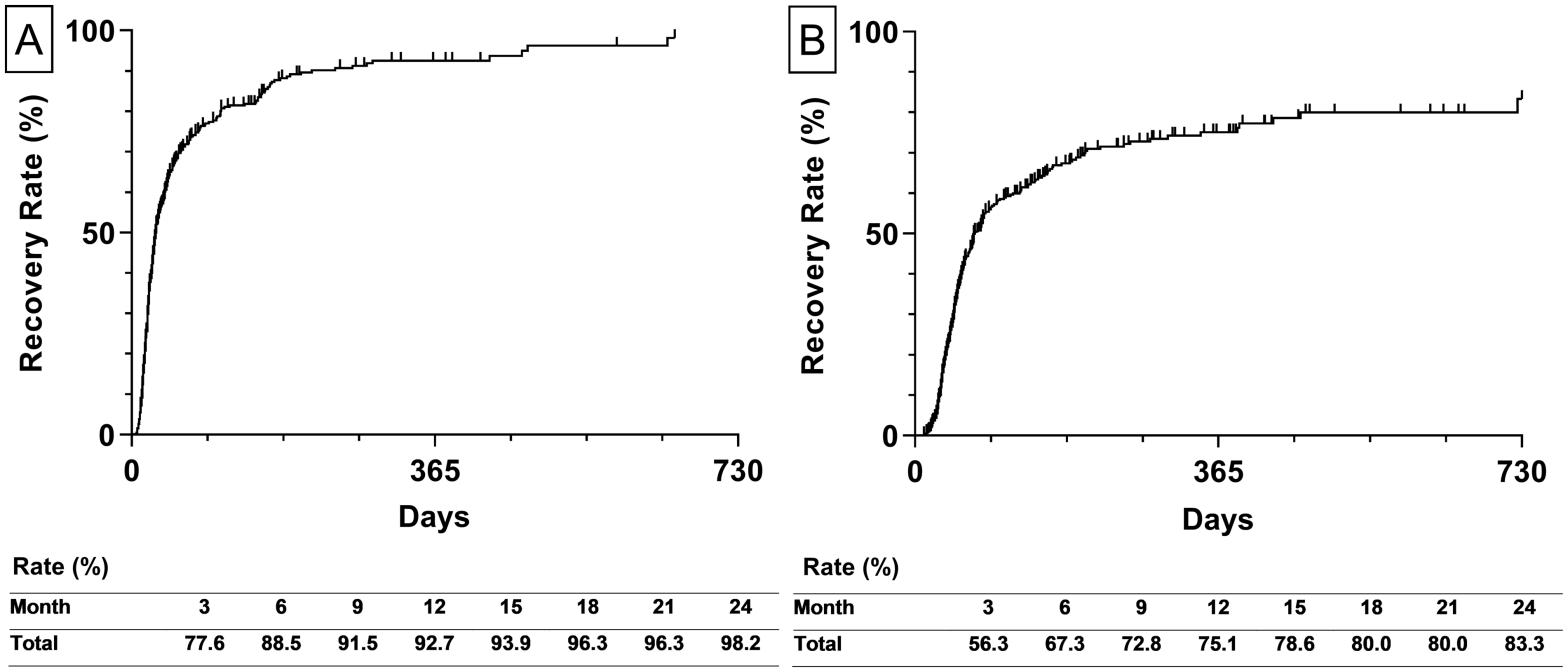
The estimated recovery rate of all participants. **(A)** The left graph depicts the estimated recovery rate to House-Brackmann Grade 2. **(B)** The right graph depicts the estimated recovery rate to House-Brackmann Grade 1. The estimated recovery rates at 3-month intervals are shown at the bottom of both graphs. The estimated recovery rates and graphs were analyzed using the Kaplan–Meier method.

The estimated recovery rate to HB Grade 2 and 1 within 2 years from the onset was represented by the Kaplan–Meier curves. The recovery rate to HB Grade 2 was 92.5% in the first year and reached 98.2% after 2 years. The median recovery time for HB Grade 2 was 29 days (interquartile range [IQR] 17–77 days; Figure 3A). The recovery rate to HB Grade 1 was 75.1% after 1 year and reached 83.3% after 2 years. The median recovery time for HB Grade 1 was 72 days (IQR 42–344 days; Figure 3B).

**Figure 4:**
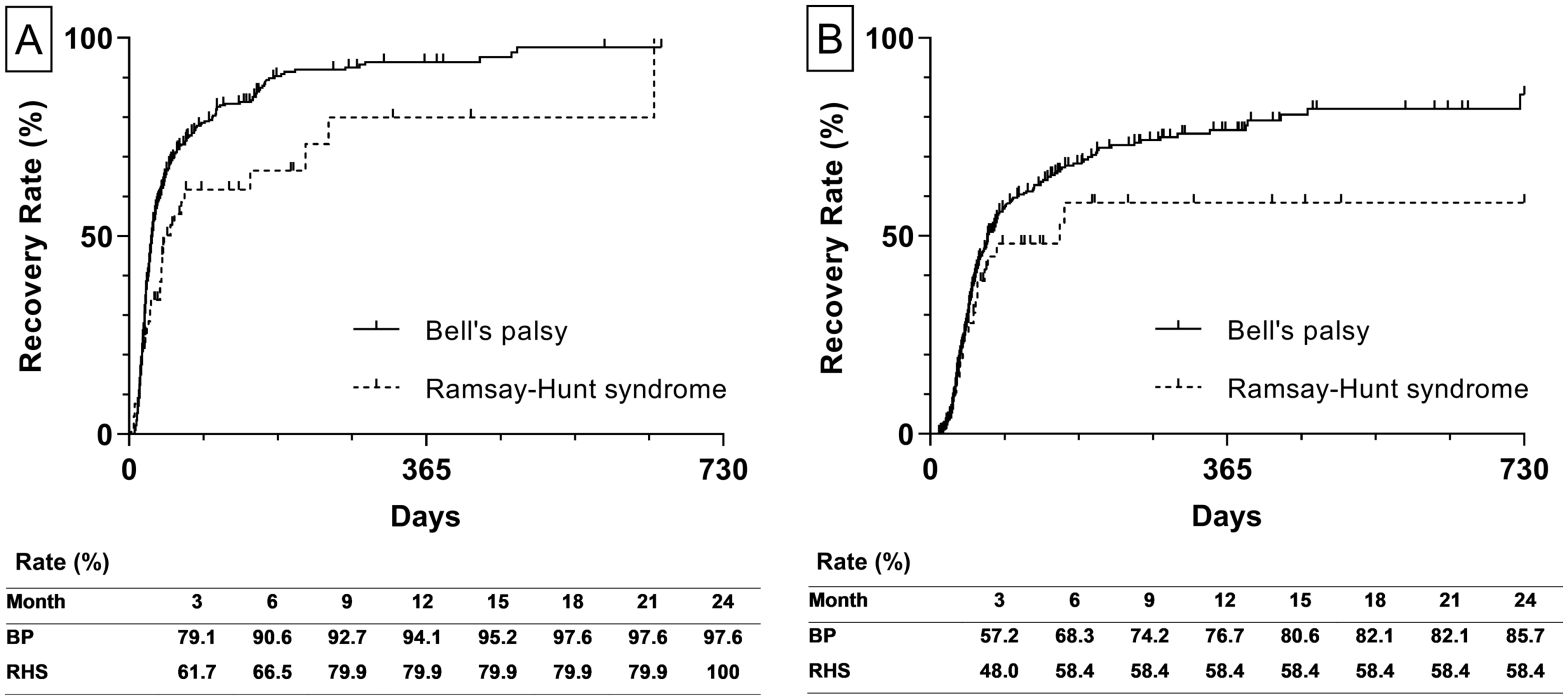
Estimated recovery rate by types of diagnosis. **(A)** The left graph depicts the estimated recovery rate to House-Brackmann Grade 2. **(B)** The right graph depicts the estimated recovery rate to House-Brackmann Grade 1. The estimated recovery rates at 3-month intervals are shown at the bottom of both graphs. The estimated recovery rates and graphs were analyzed using the Kaplan–Meier method. BP, Bell’s palsy; RHS, Ramsay-Hunt syndrome.

Additionally, the estimated recovery rate by types of diagnosis was calculated. The recovery rate to HB Grade 2 of BP and RHS was 97.6 and 100%, respectively (Figure 4A). The recovery rate to HB Grade 1 of BP and RHS was 85.7 58.4%, respectively (Figure 4B).

### 3.3 Prognostic factors associated with recovery

In order to determine the prognosis indicators related to the recovery of PFP to HB Grade 1, Cox regression analysis was performed with factors including age, sex, history of HTN and DM, HbA1c, diagnosis, palsy side, accompanying symptoms, HB Grade at onset, AAL, and MAL. Prior to multivariate analysis, univariate analysis was conducted with a reference *p*-value of 0.2 to screen the candidate factors and proposed sex, history of DM, HbA1c, diagnosis, periauricular pain, hearing impairment, and taste disorder, HB Grade at onset, AAL, and MAL as factors associated with prognosis.

Multivariate analysis performed on the selected factors indicated that AAL and MAL were significant independent predictive factors for the recovery of PFP. Higher AAL values were associated with decreased recovery rates. Relatively to the group with AAL under 60%, the groups with 60% ≤ AAL < 70, 70% ≤ AAL < 80, 80% ≤ AAL < 90%, and AAL ≥ 90% had hazard ratios of 0.655 (95% confidence interval [CI] 0.431–0.993, *p* = 0.046), 0.432 (95% CI 0.265–0.706, *p* = 0.001), 0.143 (95% CI 0.067–0.306, *p* < 0.001), and 0.085 (95% CI 0.022–0.329, *p* < 0.001), respectively. Compared with MAL under 60%, MAL values over 90% had a hazard ratio of 0.429 (95% CI 0.206–0.893, *p* = 0.024; Table 2).

**Table 2:** Cox regression analyses of 2-years recovery to HB Grade 1.

Variables		Univariate analysis			Multivariate analysis		
		Hazard ratio	95% CI	*p* value	Hazard ratio	95% CI	*p* value
Age	Years	0.995	0.987–1.003	0.232			
Sex	Male	(ref.)			(ref.)		
	Female	1.300	1.036–1.632	0.024^†^	1.245	0.980–1.582	0.073
HTN	Positive	0.844	0.641–1.113	0.230			
DM	Positive	0.721	0.521–0.998	0.049^†^	0.814	0.515–1.288	0.380
HbA1c	%	0.934	0.845–1.033	0.183^†^	1.059	0.915–1.226	0.442
Diagnosis	Bell’s palsy	(ref.)			(ref.)		
	RHS	0.721	0.472–1.103	0.132^†^	0.952	0.599–1.513	0.988
Palsy Side	Right	(ref.)					
	Left	1.064	0.853–1.327	0.584			
Periauricular pain	Positive	0.666	0.534–0.832	< 0.001^†^	0.925	0.730–1.173	0.520
Hearing impairment	Positive	0.814	0.597–1.111	0.195^†^	1.074	0.774–1.489	0.669
Taste disorder	Positive	1.280	1.018–1.608	0.034^†^	1.213	0.950–1.549	0.121
HB Grade on onset	2	(ref.)			(ref.)		
	3	0.582	0.318–1.066	0.080	0.721	0.386–1.346	0.304
	4	0.269	0.149–0.485	<0.001^†^	0.592	0.316–1.107	0.101
	5	0.127	0.068–0.239	<0.001^†^	0.519	0.263–1.025	0.059
AAL	<60%	(ref.)			(ref.)		
	60 ≤ % < 70	0.437	0.321–0.596	<0.001^†^	0.655	0.431–0.993	0.046^‡^
	70 ≤ % < 80	0.273	0.194–0.384	<0.001^†^	0.432	0.265–0.706	0.001^‡^
	80 ≤ % < 90	0.066	0.040–0.111	<0.001^†^	0.143	0.067–0.306	<0.001^‡^
	>90%	0.032	0.010–0.101	<0.001^†^	0.085	0.022–0.329	<0.001^‡^
MAL	<60%	(ref.)			(ref.)		
	60 ≤ % < 70	0.774	0.566–1.059	0.110	0.853	0.615–1.185	0.344
	70 ≤ % < 80	0.453	0.327–0.629	<0.001^†^	0.719	0.484–1.067	0.102
	80 ≤ % < 90	0.263	0.192–0.361	<0.001^†^	0.660	0.412–1.055	0.082
	>90%	0.061	0.038–0.094	<0.001^†^	0.429	0.206–0.893	0.024^‡^

Univariate Cox regression analysis was performed for each factor. Subsequently, multivariate Cox regression analysis was performed on factors that satisfy the 0.2 cut-off value in the univariate analysis. The lower value of the hazard ration indicates worse prognosis. ^†^*p* < 0.2 by univariate Cox regression analysis; ^‡^*p* < 0.05 by multivariate Cox regression analysis. CI, confidence interval; HTN, hypertension; DM, diabetes mellitus; HbA1c, hemoglobin A1c; RHS, Ramsay-Hunt syndrome; HB, House-Brackmann; AAL, average axonal loss; MAL, maximum axonal loss.

### 3.4 Survival tree analysis for the prognostic factors

**Figure 5:**
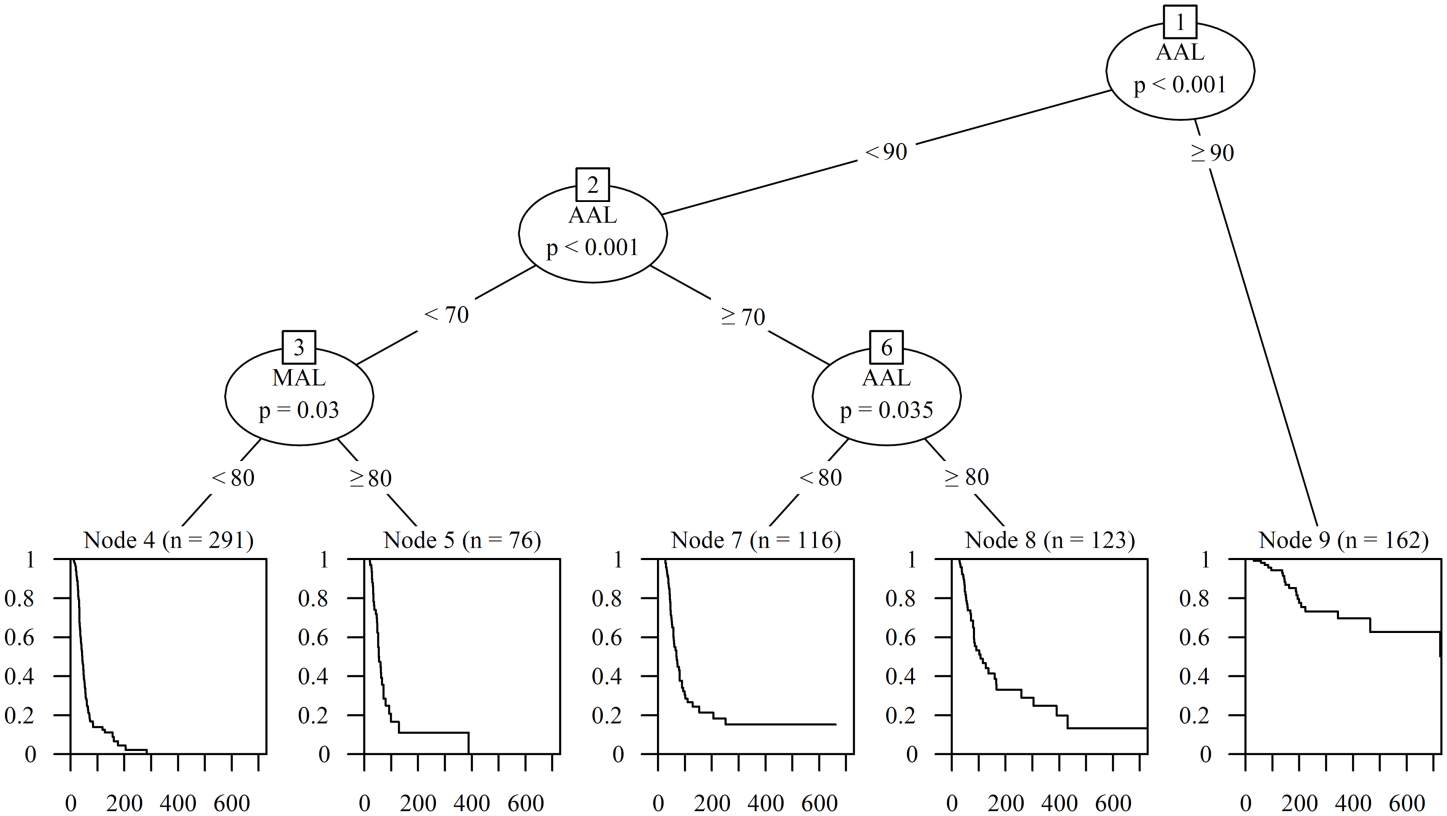
Classification according to prognosis using survival tree analysis. AAL, Average axonal loss; MAL, Maximum axonal loss.

Based on the factors derived from the Cox regression analysis, a survival tree analysis model was established for the classification of patients with PFP. Starting with the first node, the tree divided the participants based on 90% cut-off of AAL (*p* < 0.001). On the left side of the tree, the subgroup with AAL under 90% was split by AAL with a cut-off of 70% (p < 0.001). The subgroup with AAL < 70% was subsequently split by MAL with a cut-off of 80% (*p* = 0.030), and the other subgroup with 70% ≤ AAL < 90% was split by 80% cut-off of AAL (*p* = 0.035). Finally, the classification of the recovery rate of patients with PFP was determined based on five groups according to the AAL and MAL values (Figure 5).

### 3.5 Survival rate according to the classification of the survival tree analysis

**Figure 6:**
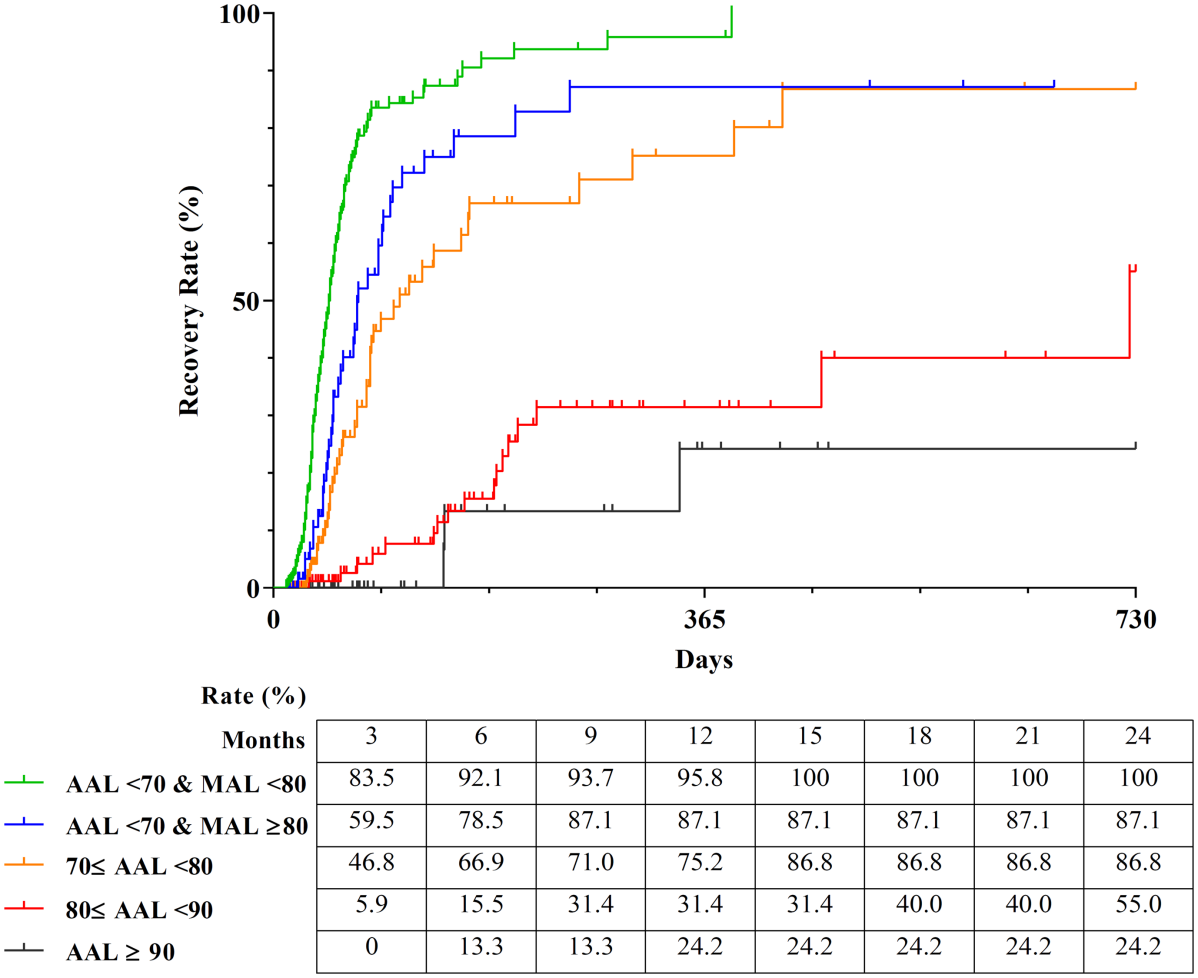
Estimated recovery rate of five classifications by survival tree analysis. The Kaplan–Meier curves shows the 2-year estimated recovery rates in each group classified by AAL and MAL. The estimated recovery rates for each group at 3-month intervals are shown at the bottom of the graph. AAL, Average axonal loss; MAL, Maximum axonal loss.

The Kaplan–Meier curves of the five groups over 2 years were obtained to calculate the detailed estimated recovery rate over time. The recovery rate to HB Grade 1 of the subgroup with AAL < 70% and MAL < 80% (*n* = 291), which exhibited the best prognosis, reached 100% at 388 days from onset. The subgroup with AAL < 70% and MAL ≥ 80% (*n* = 76) showed a superior outcome with a recovery rate of 87.1%, and the subgroup with 70% ≤ AAL < 80% (*n* = 116) also showed a good prognosis of 86.8%. The subgroup with 80% ≤ AAL < 90% (*n* = 123) showed a recovery rate of 55.0%, while the subgroup with AAL ≥ 90% (*n* = 162) showed the worst prognosis at 24.2% (Figure 6).

### 3.6 Additional analysis: survival tree analysis at initial assessment without NCS

Classification and predicting models derived from the main result can only be used after 14 days from the onset with NCS. For patients who were unable to perform an NSC or who needed early prediction at initial assessment without NCS, survival tree analysis was performed with factors including age, sex, HTN, DM, HbA1c, type of diagnosis, periauricular pain, auditory impairment, taste deficit, and HB Grade on the onset.

Starting with the first node, the tree divided the participants based on HB Grade 3 (*p* < 0.001). On the right side of the tree, the subgroup with HB Grade over 3 was split by HB Grade 4 (*p* < 0.001). Finally, the classification of the recovery rate of patients with PFP was determined based on three groups according to the HB Grade on onset.

**Figure 7:**
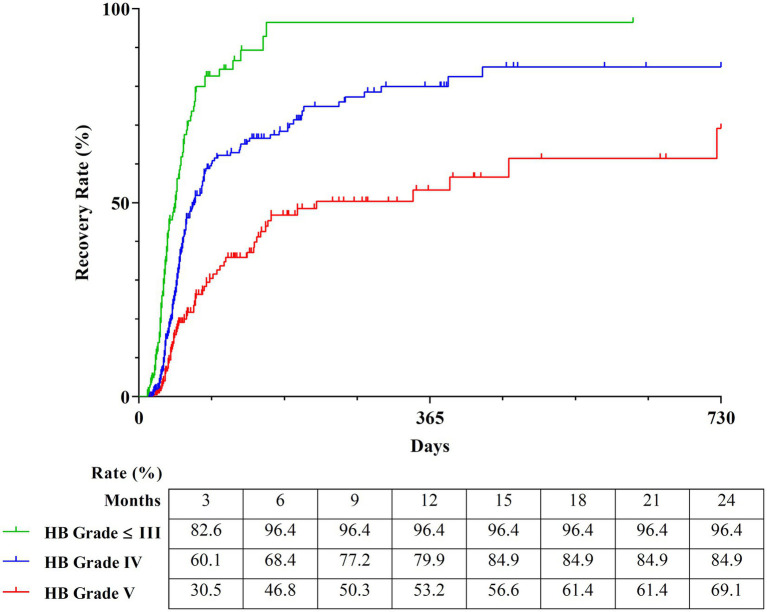
Estimated recovery rate of three classifications by survival tree analysis. The Kaplan–Meier curves show the 2-year estimated recovery rates in each group classified by HB Grade. The estimated recovery rates for each group at 3-month intervals are shown at the bottom of the graph. HB Grade, House-Brackmann Grade.

The Kaplan–Meier curves of the three groups over a period of 2 years were obtained to calculate the detailed estimated recovery rate over time. The recovery rate to HB Grade 1 of the subgroup with HB Grade 2 and 3 (*n* = 167), which exhibited the best prognosis, reached 96.4%. The subgroup with HB Grade 4 (*n* = 382) showed a good prognosis with a recovery rate of 84.9%. The subgroup with HB Grade 5 (*n* = 219) showed a recovery rate of 69.10% (Figure 7).

## 4 Discussion

This study retrospectively analyzed the medical records of 768 patients with PFP over 2 years. The estimated recovery rates of HB Grade 2 and 1 were found to be 98.2 and 83.3%, respectively, and AAL and MAL were identified as significant predictive indicators for complete recovery during the acute stage. Prognostic outcomes were significantly varied among the five classified groups based on those factors, with the estimated recovery rate of 100, 87.1, 86.8, 55.0, and 24.2%, respectively.

Previous clinical studies have investigated the recovery rates to HB Grade 2 and 1 in BP and RHS patients. Among randomized-controlled studies from systematic reviews about BP (45, 46) and RHS (47, 48) in the Cochrane Library, Sullivan et al. reported a recovery rate of 88.1% in BP with mean initial severity of HB Grade 3.6 (29). Yeo et al. showed a recovery rate of 89.0% in BP with mean severity of HB Grade 3.71 in the earlier stage (49). Derek et al. revealed the overall recovery rate of 90.2% to HB Grade 1–2 function (70.3% to HB Grade 1, 17.0% to HB Grade 2) in BP and that patients with HB Grade 5 and 4 were most likely to develop synkinesis as a sequelae of PFP (50).

In addition, among observational studies with more than 300 sample size, Peitersens et al. reported the recovery rate of 71, 21% each in BP and RHS (13). Ryu et al. suggested the recovery rate of 81.7, 58.7% in BP and RHS, whose initial severity were HB Grade 3.75 and 4.13, respectively (51). Kang et al. reported the recovery rate of 88.0% to HB Grade 1 in BP (52). The variation in recovery rates observed in both this present study and previous studies investigating the prognosis of BP and RHS is likely attributed to differences in initial severity during the early stages, which makes it difficult to compare the effectiveness of treatment accurately. Therefore, consideration of baseline characteristics including the severity of disease, is important in expecting the prognosis of PFP.

The NCS, also known as the electroneurography (ENoG), is an electrodiagnostic tool recording the compound action potential of facial muscles and evaluating the amount of nerve fiber degeneration. It has been identified as the most powerful prognostic indicator in patients with PFP, which corroborates our current findings.

Derek et al. analyzed 112 BP patients with 6-month follow-up and suggested that the chance of recovery to HB Grade 1 got worse as the greater degeneration on ENOG, whose results correlate with HB Grade after recovery (50). Munetaka et al. reported that early HB Grade and ENoG served as predictors of prognosis by researching 114 patients with BP over a 6-month period (53). Taketomo et al. conducted a research on 168 patients with BP and RHS and found that ENoG was a more reliable prognostic indicator than the initial severity assessed with HB Grade (22). Byun et al. found that the cut-off value of ENoG influencing the prognostic outcome was 82.5 and 78% in BP and RHS, respectively, after 1-year observation in 88 patients (54).

In order to establish a more accurate and clinically useful predicting model, we applied survival analysis methods, including the Cox regression model, survival tree analysis model, and Kaplan–Meier curve, using a large sample and a long-term observation period of 2 years from onset. Generally, the survival analysis method is suitable for application to a disease with a long observation period, because data from patients with follow-up loss are available for statistical analysis. In addition, since the time variable is included in the analysis together with the occurrence of the event, it is possible to derive the result value reflecting the course of time (55).

In the Cox regression model, multivariate analysis can be used to consider the interaction between the various factors that affected the outcome in each univariate analysis. Furthermore, the influence of each factor on the prognosis becomes more apparent through the hazard ratio. The survival tree model is suitable for developing a classification system for better decision-making because it presents the tree-shaped classification structure based on the significant difference according to the cut-off values of the factors (56). The Kaplan–Meier curve can provide the estimated recovery rate over time by considering the occurrence and time of the event (57). Overall, we developed a prognostic prediction model that is intuitive and easy to use in clinical practice by suggesting the estimated recovery rate based on the classification system.

In this study, to increase the homogeneity of the interventions, the participants were limited to patients hospitalized within the first 5 days from the onset and received a systematically organized IMT procedure. Since treatment in the acute stage significantly impacts the prognosis of PFP (33, 58), it is difficult to determine whether treatment was performed appropriately in patients who only visited the outpatient clinic. There is a high probability that follow-up observations on examinations will be discontinued. Therefore, patients who only visited the outpatient clinic were excluded. In addition, modern conventional treatments, including corticosteroids, antiviral agents, and physical therapy (32, 33), and TKM treatments, including acupuncture, pharmacopuncture, moxibustion, and herbal medicine (34, 35), which have been mainly suggested in guidelines and studies, were integrated to provide homogeneous treatment to patients. By providing highly homogeneous treatment to patients from the acute stage to after recovery, it was possible to analyze the effects on prognosis limited to demographic information, facial assessment, and diagnostic examination results.

Although there is a limitation in the comparison with heterogeneous data from previous studies, IMT showed favorable therapeutic effectiveness at the same time of assessment in patients with PFP. The recovery rate of IMT was significantly higher than natural progress (13). IMT showed a significantly higher recovery rate in more severe patients than treatment with corticosteroids and/or antivirals (29, 49, 52). In one study, IMT showed a similar or lower recovery rate, however, the initial HB Grade was more severe in participants of this study (51). Considering the expert responses and the progressive nature of facial palsy, various interventions in both conventional medicine and TKM need to be included in relevant critical pathways (36).

This present study, however, has several limitations. First, this was a retrospective study, which may induce a bias in the results. Second, the interval between the outpatient evaluation visits was irregular, suggesting that the evaluation point does not accurately reflect the time when the actual symptom recovery occurred. Third, as the treatment of PFP tends to be prolonged, loss of follow-up occurs frequently, which lowers the quality of the study. In addition, since the LOCF method was applied to missing evaluation records, more conservative results could have been obtained. In addition, we controlled the treatment conditions by limiting the participants to those who received the same IMT.

Comprehensively, IMT showed good clinical results in severe PFP, and further studies will be necessary to overcome the limitations of the present study and establish a higher level of evidence for IMT. Classification models of five groups based on the AAL and MAL will be helpful for clinicians to consult the prognosis and the treatment plan with their patients.

## 5 Conclusion

In conclusion, the AAL and MAL of the NCS were significant factors for predicting the prognosis of PFP. A classification model of five groups (Group 1, AAL < 70% and MAL < 80; Group 2, AAL < 70% and MAL ≥ 80%; Group 3, 70% ≤ AAL < 80%; Group 4, 80% ≤ AAL < 90%; Group 5, AAL ≥ 90%) based on the AAL and MAL may enable clinicians to consult the prognosis and the treatment plan with their patients.

## Data Availability

The raw data supporting the conclusions of this article will be made available by the authors, without undue reservation.
